# Anthropometric Changes Using a Walking Intervention in African American Breast Cancer Survivors: A Pilot Study

**Published:** 2005-03-15

**Authors:** Diane B Wilson, Jerlym S Porter, Gwen Parker, James Kilpatrick

**Affiliations:** Department of Internal Medicine and Massey Cancer Center; Department of Psychology, Virginia Commonwealth University, Richmond, Va; Massey Cancer Center, Virginia Commonwealth University, Richmond, Va; Department of Biostatistics, Virginia Commonwealth University, Richmond, Va

## Abstract

**Introduction:**

African American women exhibit a higher mortality rate from breast cancer than do white women. African American women are more likely to gain weight at diagnosis, which may increase their risk of cancer recurrence and comorbidities. Physical activity has been shown to decrease body mass index and improve quality of life in cancer survivors. This study was designed to evaluate the feasibility and impact of a community-based exercise intervention in African American breast cancer survivors.

**Methods:**

A theory-based eight-week community intervention using pedometers with scheduling, goal setting, and self-assessment was tested in a convenience sample of African American breast cancer survivors (n = 24). Data were collected at three time points to examine changes in steps walked per day, body mass index, and other anthropometric measures, attitudes, and demographic variables.

**Results:**

Statistically significant increases in steps walked per day and attitude toward exercise as well as significant decreases in body mass index, body weight, percentage of body fat, and waist, hip, and forearm circumferences, as well as blood pressure, were reported from baseline to immediate post-intervention. Positive changes were retained or improved further at three-month follow-up except for attitude toward exercise. Participant retention rate during eight-week intervention was 92%.

**Conclusion:**

Increasing walking for exercise, without making other changes, can improve body mass index, anthropometric measures, and attitudes, which are associated with improved quality of life and reduced risk of cancer recurrence. The high participant retention rate, along with significant study outcomes, demonstrate that among this sample of African American breast cancer survivors, participants were motivated to improve their exercise habits.

## Introduction

African American women exhibit a higher rate of breast cancer mortality when compared with white women (1,2). Being diagnosed later, variation in treatment response, and larger tumor size have all been identified as factors that may contribute to differences in breast cancer survival time (3). Obesity is also more prevalent among African American women. Although the majority of women report weight gain after breast cancer diagnosis, African American women are at greater risk for this pattern (4). Being overweight is not only associated with increased risk of cancer recurrence but also with comorbid conditions such as heart disease, stroke, diabetes, and depression, all of which may contribute to decreased quality of life and shorter survival time (5-10). By contrast, being more physically active is associated with improved quality of life and decreased body mass index (BMI) in cancer survivors, which in turn may contribute to longer survival time (11,12).

Until recently, women who have completed cancer therapy have been offered little to improve survival or decrease risk of new disease. Yet studies show that as a group, breast cancer survivors are interested in improving their health behaviors and quality of life (4,7). Two randomized trials (13,14) are currently testing healthy lifestyle interventions for cancer survivors. However, very few interventions have been developed and tested specifically among African American women with breast cancer, even though they are a population at high risk for recurrence and comorbid disease.

To address weight gain in African American breast cancer survivors, we designed a theory-based cognitive-behavioral walking program to test its feasibility and impact on steps per day and BMI. The study was pilot tested among African American breast cancer survivors, using a community education model in an urban inner-city setting.

## Methods

We pilot tested an eight-week community-based walking program in a convenience sample of African American breast cancer survivors (n = 24) to investigate feasibility and impact on outcome measures over three time points: 1) baseline, 2) immediate post-intervention, and 3) three-month follow-up. African American women who 1) had been diagnosed with breast cancer, 2) had completed treatment at least three months before recruitment, 3) were mobile, and 4) were less than 70 years of age were eligible for the study.

### Participant recruitment

Using a broad, organized effort, participants were recruited from Massey Cancer Center clinics, outreach sites, contacts with local churches, community leaders, and breast cancer organizations including breast cancer support groups. A city council member, along with other breast cancer survivors, was also instrumental in communicating study information throughout the community. Flyers, television announcements, and personal communication were used during the three-month recruitment effort. We contacted approximately 230 potentially eligible women. Recruitment rate was approximately 10%. Reasons for nonparticipation included having cancer treatment within the prior three months, not being able to attend community meetings because of work or family commitments, or having comorbid conditions that decreased mobility.

### Intervention

The theory-driven intervention was designed with the primary study goal of integrating walking into one's daily routine. The Health Belief Model was used as the theoretical framework for the intervention (15). This well-known model is based on perceived seriousness and perceived susceptibility as the strongest predictors for the implementation of health behaviors. Thus, the intervention was designed on the basis that breast cancer survivors are a population who have experienced a serious disease and perceive their susceptibility toward a cancer recurrence. Eight 75-minute weekly sessions were held at a community center (evening) and at a local church (noontime). Sessions were presented by the same instructor and staff, using a curriculum that described benefits and barriers to exercise, its relationship to health and cancer risk, and personal assessment/problem-solving sessions for motivation. Didactic, interactive, and small-group processes were used during each session. Steps-only pedometers were tested, and progressive step goals were provided. Participants scheduled walking times for the upcoming week and reported steps walked per day for the previous week using scheduler/tracker forms. Patients served as their own controls.

### Study variables

Study variables were assessed at three time points: baseline, immediate post-intervention, and at three-month follow-up. The study goal was the integration of walking into the participant's daily routine. The primary study outcomes were changes in number of steps and BMI. Steps per day were measured using a steps-only pedometer. Participants were instructed to wear the pedometer upon rising in the morning until bedtime and to record the number of steps walked. BMI was calculated from weight and height using a calibrated scale. Waist, hip, and upper arm circumferences were measured using a tape measure, and blood pressure was measured with a standard blood pressure cuff. Body-fat percentage was measured using Futrex, a portable near-infrared sensor system (16). All clinical measures were taken by a clinical nurse practitioner.

Participant demographic information, cancer history, and attitudinal measures were assessed using standardized survey items from other study instruments. The instrument was pilot tested in a comparable age group of African American women. Attitudes toward exercise were measured using the Exercise Decisional Balance instrument (17), a 16-item questionnaire focused on avoidance of exercise (cons) and positive perceptions of exercise (pros). Cancer anxiety was measured using the Cancer Anxiety Scale (18), and participants' concern about cancer recurrence was assessed.

### Data collection and statistical analysis

Data were entered into a database using SPSS statistical software (SPSS Inc, Chicago, Ill). Descriptive statistics were determined for all study variables. Analysis of variance was performed to test for differences in measures collected at baseline, immediately after intervention, and at three-month follow-up. Paired *t* tests were used to determine differences in mean anthropometric and attitudinal measures between the three time points. In addition, based on frequency distribution of time since diagnosis, all study variables were tested among those diagnosed three years or less prior to start of the intervention (1999–2002) (n = 10) and those diagnosed earlier (1978–1998) (n = 12), using independent samples *t* tests**.**

## Results

Twenty-four women were enrolled in the intervention study. One participant dropped out because of scheduling conflicts. One experienced a cancer recurrence, resulting in 22 eligible women completing the intervention.

Table 1 shows the characteristics of the study sample. Mean age was 55 years (range 47–66 years). The majority of the women had post-high school education. The sample was approximately 50% married and 50% divorced, widowed, or single. Receiving both chemotherapy and radiation therapy was most prevalent among participants (46%), with 18% receiving radiation alone, chemotherapy alone, or neither treatment; 23% were currently taking tamoxifen. Forty-five percent of participants (10/22) had been diagnosed with breast cancer in or since 1999, and 55% (12/22) were diagnosed before 1999. For most participants (91%), this was their first cancer diagnosis.

### Feasibility

Feasibility was determined by examining attendance at weekly sessions, study retention, and receptivity to pedometer use. Attendance at weekly sessions was excellent, with 70% of the participants attending seven or more intervention sessions. Study retention to the eight-week study was also excellent, with 22 of 24 women completing the intervention and immediate post-assessment. Participants had positive experiences using the pedometers and recording steps per day. Broken or lost pedometers were reported by approximately 25% of the study sample, and they were replaced to ensure continuous data collection. Additional data showed that 95% responded "about right" to a survey item asking whether number of study sessions were too many, too few, or about right.

### Impact on study outcomes

Results of ANOVA analyses of repeated measures (baseline, immediate post-intervention, and at three-month follow-up) showed statistically significant differences in steps per day (*P* < .001), hip circumference (*P* = .009), forearm circumference (*P* < .001), systolic blood pressure (*P* = .002), diastolic blood pressure (*P* = .001), and attitude toward exercise (*P* = .005).

Table 2 shows the difference in mean study measures using paired *t *tests, from baseline to immediate post-intervention. Mean steps per day significantly increased from 4791 to 8297 (*P* < .001). Other significant decreases included the following: BMI (*P* = .004), body weight (*P* = .005), percentage body fat (*P* = .003), and forearm circumference (*P* = .007). Increased positive perception of exercise was also reported (*P* = .03).

Table 3 shows study results among women who completed the three-month follow-up assessment (n = 17). From immediate post-intervention to the three-month follow-up, mean steps per day did not significantly change. There were statistically significant improvements in hip circumference (*P* = .04), forearm circumference (*P* = .04), and diastolic blood pressure (*P* = .02). Thus, all anthropometric measures either stayed the same or showed further improvement by further reduction in measures from immediate post-intervention to three-month follow-up. Of all study variables, only attitude toward exercise significantly changed direction (*P* < .001), with women showing a more negative opinion of exercise by three-month follow-up compared with immediate post-intervention. There were no differences in mean study outcomes in the participants who did not attend three-month follow-up assessment sessions (n = 5) compared with participants who did attend and had measurements (n = 17).

### Time since diagnosis

More recently diagnosed women tended to have higher body measures at all three time points, but only diastolic blood pressure was significantly higher at baseline (*P* = .02) when compared to earlier diagnosed women. The same effect was true at immediate post-intervention for both diastolic blood pressure (*P* = .02) and systolic blood pressure (*P* = .003). At three-month follow-up, recently diagnosed women were significantly more likely to have higher waist measures (*P* = .048), with trends toward larger hip (*P* = .06) and body fat (*P* = .05) measures than earlier diagnosed women.

## Discussion

We found statistically significant changes in the main study outcomes of steps per day, BMI, and virtually all of the anthropometric changes measured in the study population after an eight-week intervention, with most results remaining at three-month follow-up. The breast cancer survivor participants were motivated and compliant with the intervention, which likely enhanced their success. Having had cancer and understanding their risk of recurrence may account for the strong motivation we found in this population, as suggested by the constructs of the Health Belief Model. It is also important to note that the sample participants all had more than a high school education, which may also have contributed to their success.

Although we found only a few statistically significant differences in mean body measures in relationship to time since diagnosis, we may have detected more evidence of this pattern had we had a larger study sample. While not significant, the more recently diagnosed women had larger body measures and lower mean steps per day than earlier diagnosed women at both immediately post and at three-month follow-up.

The goal of the study was to have women integrate walking into their daily routines on their own. They attended sessions for education, motivation, and self-assessment; walking did not take place during the study sessions. This was an important feature of the study design because research shows that compliance is likely to decline significantly after an intervention is completed (19). Thus, our results showing that mean steps per day stayed relatively steady even at three-month follow-up was encouraging.

### Anthropometric and clinical measures

The mean change in body weight was modest but significantly less than baseline. This level of weight loss supports what similar interventions have reported (20). We were encouraged to see weight loss occur among participants using an exercise-only intervention. Nearly every participant posted decreases in at least one anthropometric measure, so that even among women who did not show weight loss, decreases were noted in body circumferences or blood pressure. We were also encouraged to see that anthropometric improvements did not fall off at three-month follow-up, and some improved further. It is possible that adding dietary modifications to the exercise intervention would contribute to more substantial anthropometric changes.

### Attitudinal factors

Women in the study improved their attitude toward exercise from baseline to post-intervention by reporting fewer barriers to exercise over the study period. This was not surprising given the focus of the intervention sessions on overcoming personal obstacles to exercise. However, the attitudinal improvement did not hold at three-month follow-up. Although steps per day did not significantly change at three-month follow-up, one might wonder if the decline in exercise attitude might eventually negatively influence exercise behavior after a longer time interval. Cancer stress scores did not change significantly over the course of the intervention. However, scores for this variable were not particularly high even at the start of the intervention. This could be related to the fact that only 18% of participants were diagnosed during the 12 months prior to the start of the study. Cancer stress may subside as time passes after a woman's diagnosis. Had we studied a group of more recently diagnosed women, we may have found more evidence of cancer stress at baseline and potential for impact after the exercise intervention than we did the with this study population.

### Limitations

This study was limited by the study size and lack of control group. For a pilot study, however, the sample size was adequate to study feasibility and study outcomes. In addition, the study reflects the common limitations for relying on self-report data. Anthropometric variables were included in the study in addition to self-reported data to provide measured data for evaluating results. The intervention tested exercise only; thus, even more significant changes among this population are possible if both energy balance components — food intake *and* physical activity — are modified. Overall, the study objectives were realized, and the study provides interesting pilot data for testing a more comprehensive lifestyle intervention in a similar population**.** However, the sample does not constitute a representative sample, and the study findings may not be applicable to other breast cancer survivors.

### Summary and conclusions

Steps walked per day, BMI, body circumferences, blood pressure, and attitudinal variables all showed improved mean statistically significant changes in this population of African American breast cancer survivors after a theory-based cognitive-behavioral community intervention. The study showed strong feasibility measures in positive response to using pedometers, high participant retention, social support, and excellent compliance after eight weeks. Given data indicating obesity is associated with shorter breast cancer survival time, these study results may position breast cancer survivors to have both improved quality of life and reduced risk of cancer recurrence. Further study is needed to test a randomized comprehensive diet and exercise intervention in African American breast cancer survivors against controls in a longer, larger randomized trial with additional study variables.

## Figures and Tables

**Table 1:** Characteristics of African American Breast Cancer Survivors Participating in Eight-Week Walking Intervention (n = 22)

	**Mean (range)**
Age (yrs)	55 (47-66)
Weight (lbs)	191 (142-271)
Body mass index (kg/m^2^)	32.7 (25.2-47.2)

	**No. (%)^a^**

**Education**	

<High school	1 (4.5)
High school graduate	1 (4.5)
>High school	20 (91)

**Marital status**	

Married	11 (50)
Single/divorced/widowed	11 (50)

**Menopausal status**	

Premenopausal	3 (14)
Postmenopausal	19 (86)

**Time since diagnosis, years**	

<1	3 (14)
1-3	7 (32)
4-6	4 (18)
7-9	3 (14)
⩾10	5 (23)

**Type of treatment**	

Chemotherapy	4 (18)
Radiation therapy	4 (18)
Both	10 (46)
Neither	4 (18)
Takes tamoxifen	5 (23)
Drinks alcohol	6 (27)
Smokes	2 (9)

a Percentages do not total 100 because of rounding.

**Table 2:** Change in Group Anthropometric Measures From Baseline to Immediately After Eight-Week Walking Intervention Among African American Breast Cancer Survivors (n=22)

	**Baseline**	**Change**	***P* a**
Steps/day	4791	+3506	<.001
Body mass index (kg/m^2^)	32.7	−0.38	.004
Weight (lbs)	191.2	−2.0	.005
Body fat (%)	40.1	−3.4	.003
Waist circumference (cm)	99.3	−4.6	.04
Hip circumference (cm)	117.9	−2.1	.02
Forearm circumference (cm)	34.8	−1.5	.007
Systolic blood pressure (mm Hg)	140.9	−10.1	<.001
Diastolic blood pressure (mm Hg)	80.1	−6.2	.005
Waist-to-hip ratio	0.84	−0.02	.16
Exercise attitude total^b^	66.2	+3.0	.03
Cancer anxiety total^c^	6.8	−0.36	.20

a Paired t test for difference in group means.

b Attitudes toward exercise were measured using the Exercise Decisional Balance instrument (17).

c Cancer anxiety was measured using the Cancer Anxiety Scale (18).

**Table 3:** Change in Group Anthropometric Measures From Immediately After Eight-Week Walking Intervention to Three-Month Follow-up Among African American Breast Cancer Survivors (n=17)

	**Immediate Post-Intervention**	**Change**	***P* a**
Steps/day	8223	+22	.97
Body mass index (kg/m^2^)	31.5	+0.14	.32
Weight (lbs)	182.2	+0.62	.47
Body fat (%)	36.1	+2.1	.06
Waist circumference (cm)	95.2	−0.35	.70
Hip circumference (cm)	115.8	−1.9	.04
Forearm circumference (cm)	33.3	−0.91	.04
Systolic blood pressure (mm Hg)	129.9	−1.1	.74
Diastolic blood pressure (mm Hg)	74.1	−4.2	.02
Waist-to-hip ratio	0.82	+0.01	.24
Exercise attitude total^b^	68.6	−4.45	<.001
Cancer anxiety total^c^	6.4	−0.06	.88

a Paired t test for difference in group means.

b Attitudes toward exercise were measured using the Exercise Decisional Balance instrument (17).

c Cancer anxiety was measured using the Cancer Anxiety Scale (18).
